# Suppression of ZBP1-mediated NLRP3 inflammasome by the tegument protein VP22 facilitates pseudorabies virus infection

**DOI:** 10.1128/mbio.01945-24

**Published:** 2024-10-30

**Authors:** Zicheng Ma, Depeng Liu, Wandi Cao, Lei Guo, Kesen Liu, Juan Bai, Xingyi Li, Ping Jiang, Xing Liu

**Affiliations:** 1 Key Laboratory of Animal Diseases Diagnostic and Immunology, Ministry of Agriculture, MOE International Joint Collaborative Research Laboratory for Animal Health & Food Safety, College of Veterinary Medicine, Nanjing Agricultural University, Nanjing, China; 2 Jiangsu Co-Innovation Center for Prevention and Control of Important Animal Infectious Diseases and Zoonoses, Yangzhou, China; 3 School of Computer Science, Northwestern Polytechnical University, Xi'an, Shanxi, China; Stony Brook University, Stony Brook, New York, USA

**Keywords:** ZBP1, NLRP3 inflammasome, pseudorabies virus, VP22

## Abstract

The interaction between Z-DNA binding protein 1 (ZBP1) and the NLR family pyrin domain-containing 3 (NLRP3) inflammasome has been uncovered in several viral infections. However, the role of this molecular pathway during infection with the alpha-herpesvirus pseudorabies virus (PRV) remains largely elusive. Here, we report that during PRV infection, ZBP1-mediated NLRP3 inflammasome activation is inhibited by the viral tegument protein VP22, thereby facilitating viral infection. Through a combination of RNA sequencing and genetic studies, we demonstrate that PRV VP22 functions as a virus-encoded virulence factor by evading the inhibitory effects of ZBP1 on virus infection. Importantly, the replication and pathogenicity of a recombinant PRV lacking VP22 are significantly increased in ZBP1-deficient cells and mice. Mechanistically, PRV VP22 interacts with ZBP1, impeding the recruitment of receptor-interacting protein kinase 3 and Caspase-8, thereby inhibiting NLRP3 activation. Furthermore, we show that the N-terminal 1–50 amino acid domain of VP22 dominantly destabilizes ZBP1-mediated function. Taken together, these findings identify a functional link between PRV infection and ZBP1-mediated NLRP3 inflammatory response, providing novel insights into the pathogenesis of PRV and other herpesviruses.

**IMPORTANCE:**

Z-DNA binding protein 1 (ZBP1) functions as a pivotal innate immune sensor that regulates inflammatory cell death during viral infections. However, its role in pseudorabies virus (PRV) infection remains unknown. Here, we demonstrate that ZBP1 serves as a restrictive factor by triggering the activation of the NLR family pyrin domain-containing 3 inflammasome, a process counteracted by PRV-encoded protein VP22. Furthermore, VP22 interferes with the interaction between ZBP1 and receptor-interacting protein kinase 3/Caspase-8, particularly through its N-terminal 1–50 amino acids. Importantly, deficiency in ZBP1 enhances the replication and virulence of recombinant viruses lacking VP22 or its N-terminal 1–50 amino acids. These findings reveal how PRV escapes ZBP1-mediated inflammatory responses during infection, potentially informing the rational design of therapeutic interventions.

## INTRODUCTION

Z-DNA binding protein 1 (ZBP1), also known as DNA-dependent activator of interferon (IFN)-regulatory factors (DAI), is a key signaling initiator in the innate immune response and PANoptosis (1). As a cytosolic sensor for Z-form nucleic acids (Z-NA), ZBP1 detects both exogenous and endogenous nucleic acids. When sensing self Z-NA or harmful stimuli, ZBP1 activates nuclear factor kappa B (NF-κB) and interferon regulatory factor 3 (IRF3) signaling pathways and promotes the assembly of the ZBP1-PANoptosome (2–5). Mechanistically, the formation of the ZBP1-PANoptosome involves the recruitment of the NLR family pyrin domain-containing 3 (NLRP3) inflammasome [NLRP3, apoptosis-associated speck-like protein containing a CARD (ASC), and Caspase-1], receptor-interacting protein kinase 3 (RIPK3), Caspase-8, and Caspase-6. After PANoptosome formation, Caspase-1 cleaves pro-IL-1β and pro-IL-18, as well as gasdermin D, to release its pore-forming N-terminal structural domain (N-GSDMD). Additionally, Caspase-3 and Caspase-7 are activated to cleave gasdermin E (GSDME), which releases its pore-forming N-terminal structural domain (N-GSDME), and MLKL is phosphorylated. These events lead to membrane pore formation and release of IL-1β and IL-18, among other cytokines and DAMPs (6, 7).

Emerging evidence demonstrates that ZBP1 plays an important role during infections with different viruses. In Influenza A virus (IAV) infection, ZBP1 recognizes viral nucleic acids, triggers PANoptosis, and inhibits viral replication. However, this inflammatory process causes pulmonary cell death, resulting in mortality of infected mice due to pulmonary dysfunction and inflammation (8–10). In Severe Acute Respiratory Syndrome Coronavirus 2 (SARS-CoV-2) infection, ZBP1 upregulation via IFN-I intervention at early stages induces appropriate death of infected cells, aiding in a mild inflammatory response to clear the virus. In contrast, later IFN administration triggers ZBP1-mediated extensive inflammatory cell death in the lungs, leading to a cytokine storm, lung dysfunction, and increased mortality (4, 5, 11). For the well-known alpha-herpesvirus, Herpes simplex virus-1 (HSV-1), a large PANoptosome, forms in infected mouse bone marrow-derived macrophages (BMDMs), comprising components such as absent in melanoma 2 (AIM2), ZBP1, Pyrin, ASC, CASP1, RIPK3, MLKL, RIPK1, FADD, and CASP8. This PANoptosome efficiently suppresses HSV-1 replication and protects infected animals from death by inducing apoptosis, pyroptosis, and necroptosis (12).

Pseudorabies virus (PRV), an alpha-herpesvirus, can infect various animal species and has recently been reported to cause acute encephalitis in humans (13–17). Previous research has shown that PRV infection triggers an inflammatory response characterized by IL-1β secretion, which causes cell and tissue damage (18, 19). However, precise underlying mechanisms are unclear. In this study, we revealed that PRV VP22, a viral virulence factor, interacted with ZBP1 and suppressed ZBP1-mediated activation of the NLRP3 inflammasome, thereby facilitating viral infection. These findings offer new insights into the pathogenesis of PRV and other herpesviruses, potentially providing a foundation for future therapy.

## RESULTS

### The tegument protein VP22 contributes to PRV infection

**Fig 1:**
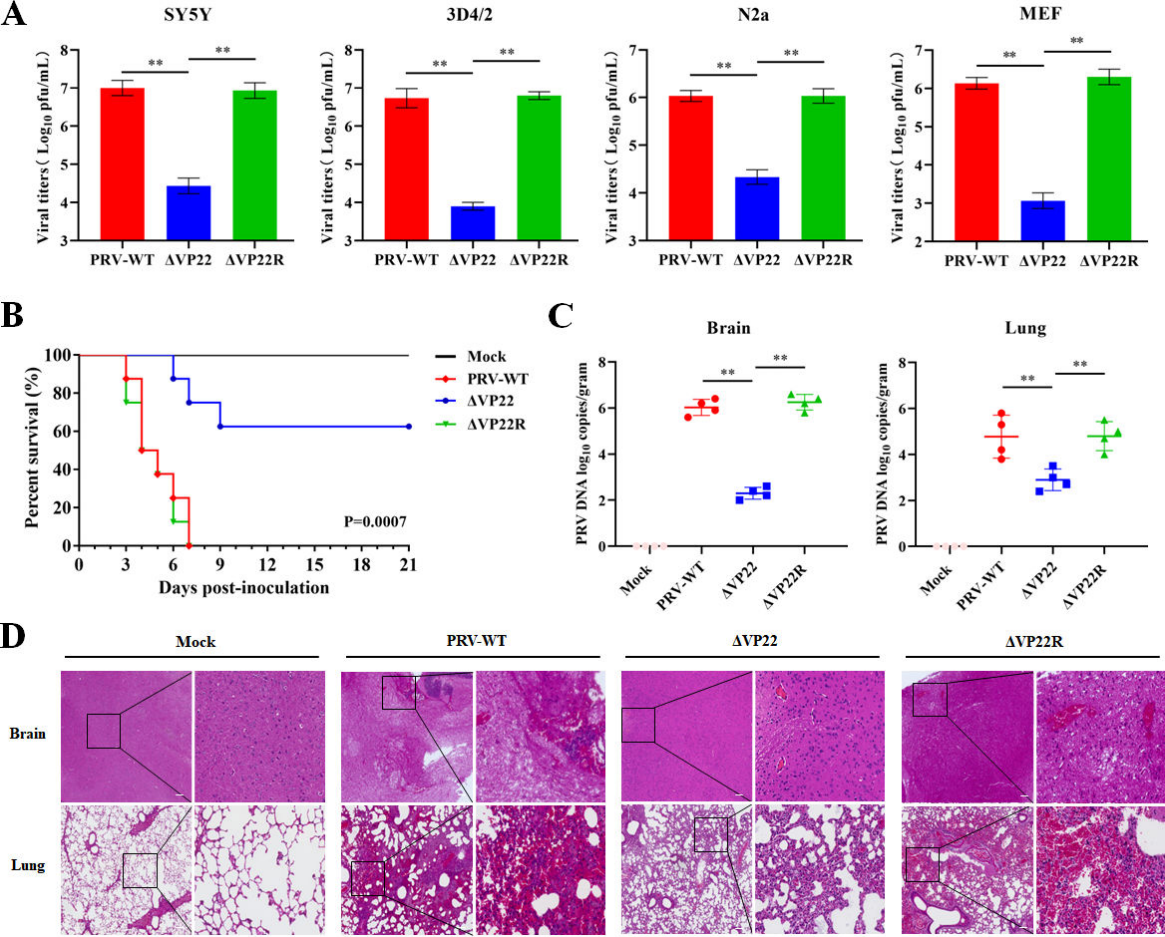
PRV VP22 promotes viral replication and pathogenicity. (**A**) Viral replication in SH-SY5Y, 3D4/2, N2a, and MEF cells. Cells were infected with PRV-WT, ΔVP22, or ΔVP22R at 0.01 multiplicity of infection (MOI). At 48 hours post-infection (hpi), cells were harvested, and the total virus titers were determined by plaque assay in Vero cells. (**B**) Monitoring of mouse survival upon infection with different PRVs, shown as percentage of live mice over time. Four experimental groups (eight mice/group), infected through subcutaneous injection, were compared: mock infected or infected with PRV-WT, ΔVP22 or ΔVP22R (1 × 10^5^ PFU/mice). Simple survival analyses for the four groups using Kaplan–Meier curves (*P* = 0.0007). (**C**) Four groups of mice (four mice/group) were treated as in (**B**) and sacrificed at 3 days post-infection (dpi). Viral DNA loads in the brain and lung were quantified by Real-Time Polymerase Chain Reaction (RT-PCR). (**D**) Brain and lung sections from mice infected with different PRVs (1 × 10^5^ PFU/mice) were analyzed by hematoxylin and eosin (H&E) staining at 3 dpi. The magnified area of each section (right-hand side) is localized by a square on the corresponding lower magnification picture (left-hand side) (scale bar: 100 µm). The data are representative of at least three independent experiments with similar results (mean ± SD of *n* = 3 biological replicates in A; *n* = 8 biological replicates in B; *n* = 4 biological replicates in C). **P < 0.01 (two-tailed unpaired t test).

VP22, a conserved tegument protein encoded by the *UL49* gene in alpha-herpesviruses, is essential for infection by HSV-1 (20). With only 26% of amino acid homology between PRV’s and HSV-1’s VP22 (Fig. S1), to delve into the role of VP22 is crucial to understand its meaning for PRV virulence. To this aim, we generated a VP22 null PRV mutant (ΔVP22) and its revertant virus (ΔVP22R) using the CRISPR-Cas9 system (Fig. S2). The consequence of these mutations was investigated by comparing viral titers obtained in SY5Y, 3D4/2, N2a, and mouse embryonic fibroblast (MEF) cell lines with the different mutants. Intriguingly, ΔVP22 titers were decreased by approximately 2 logs or more compared with those of wild-type PRV (PRV-WT) or ΔVP22R (Fig. 1A). In mouse, ΔVP22 displayed significantly reduced pathogenicity and lethality compared with PRV-WT and ΔVP22R (Fig. 1B). Correspondingly, PRV DNA loads in the brain and lung tissues of mice infected with ΔVP22 were notably lower than those in mice infected with PRV-WT or ΔVP22R (Fig. 1C). Further, neuropathological brain lesions, inflammatory cell infiltration, and lung congestion in mice infected with ΔVP22 were noticeably milder than those in mice infected with PRV-WT and ΔVP22R (Fig. 1D). These findings collectively suggested that PRV VP22 plays a critical role in viral replication and pathogenicity.

### PRV VP22 inhibits interferon α/γ and inflammatory responses

**Fig 2:**
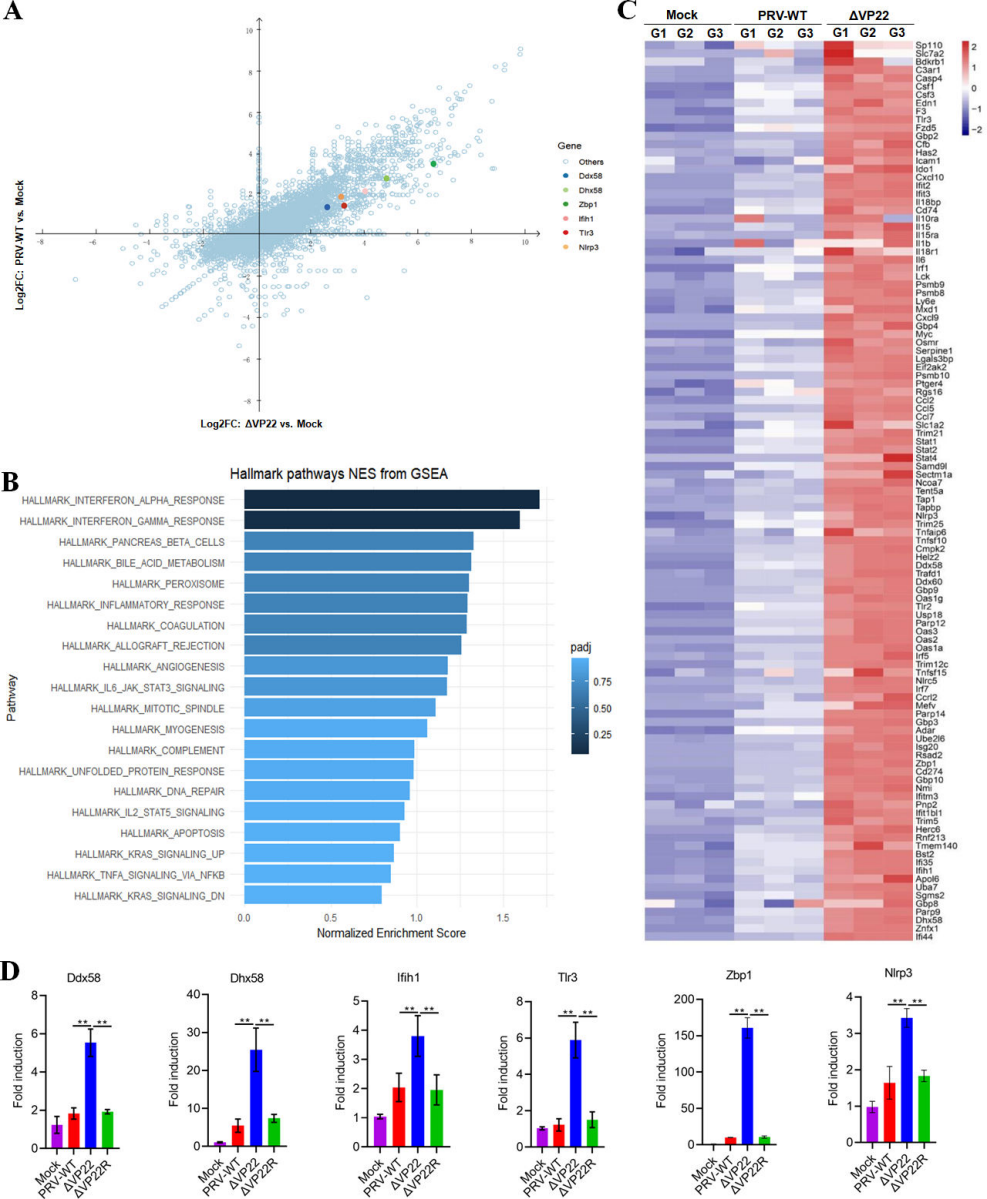
PRV-WT and ΔVP22 induce differential gene expression in infected MEF cells. (**A**) Host’s gene expression alteration analyzed by RNA-deep sequencing (RNA-seq). The dot plot shows the genes induced by ΔVP22 compared with mock RNA-Seq, versus those induced by PRV-WT compared with mock RNA-Seq. Genes encoding PRRs of interest, i.e., *Zbp1*, *Ddx58*, *Dhx58*, *Ifih1*, *Tlr3*, and *Nlrp3* are highlighted by colored dots. (**B**) GSEA of the upregulated or downregulated mRNAs. (**C**) Heatmap showing the relative expression levels of genes of the IFN-α and IFN-γ pathways and inflammatory response involved in mock and PRV-WT or ΔVP22-infected cells. (**D**) MEF cells were infected with PRV-WT, ΔVP22, or ΔVP22R at MOI of 5; at 12 hpi, the cells were harvested for RNA extraction and RT-PCR quantification of *Ddx58*, *Dhx58*, *Ifih1*, *Tlr3*, *Zbp1*, and *Nlrp3* mRNAs, and 18s rRNA levels. The data are representative of at least three independent experiments with similar results (mean ± SD of *n* = 3 biological replicates for D). **P < 0.01 (two-tailed unpaired t test).

To gain insights into the mechanism by which PRV VP22 promotes viral infection, we conducted RNA deep sequencing to compare global gene expression in MEFs responding to PRV-WT or ΔVP22 infection (Fig. 2A). Building upon this initial assessment, we sought to delineate the functional pathways associated with the genes expressed differentially between PRV-WT and ΔVP22 groups. Gene set enrichment analysis (GSEA) identified distinct pathways that were enriched, with three innate immunity-related pathways ranking in the top six: interferon-α (IFN-α), IFN-γ, and inflammatory response (Fig. 2B; Fig. S3). Furthermore, the heatmap analysis revealed an enrichment in genes of the pathways of IFN-α, IFN-γ, and inflammatory response, demonstrating significantly increased expression of these genes in ΔVP22-infected cells compared with the mock or PRV-WT-infected cells (Fig. 2C). Notably, pattern recognition receptor (PRR) genes were enriched, including *Ddx58* (*RIG-I*), *Dhx58* (*LGP2*), *Ifih1* (*MDA5*), *Tlr3*, *Zbp1*, and *Nlrp3*. The differential expression of these genes was further confirmed by quantitative real-time (qPCR) (Fig. 2D). These results suggested that VP22 promotes viral infection by inhibiting the pathways related to innate immunity.

### PRV VP22 overcomes host’s ZBP1-mediated viral inhibition

**Fig 3:**
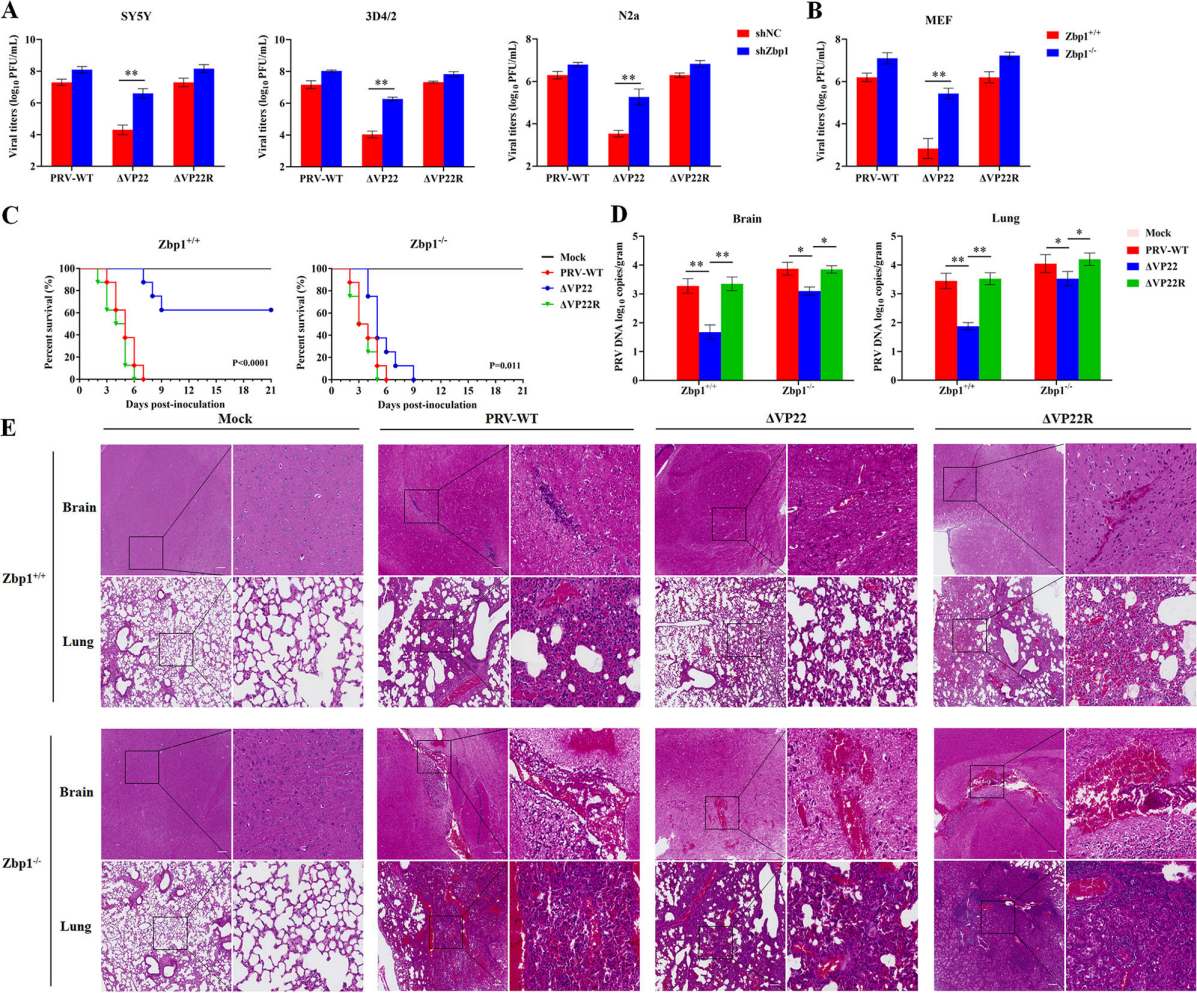
ZBP1 inhibits PRV viral replication and pathogenicity. (**A and B**) Effects of ZBP1 on PRV viral replication. (**A**) WT SH-SY5Y, 3D4/2, N2a cells and corresponding *Zbp1*-knockdown cells or (**B**) *Zbp1*^+/+^ (WT) and *Zbp1*^−/−^ MEFs were infected with PRV-WT, ΔVP22, or ΔVP22R at 0.01 MOI. At 48 hpi, cells and supernatants were harvested, and total virus titers were determined by plaque assay in Vero cells. (**C**) Comparison of the survival curves obtained for *Zbp1*^+/+^ (WT) and *Zbp1*^−/−^ mice, which were either mock infected or infected with 1 × 10^5^ PFU/mice of PRV-WT, ΔVP22, or ΔVP22R by subcutaneous injection (eight mice/group). Simple survival analyses for the four groups using Kaplan–Meier curves (*Zbp1*^+/+^: *P* < 0.0001; *Zbp1*^−/−^: *P* = 0.011). (**D**) Four groups of mice (four mice/group) treated as in **C** were sacrificed at 3 dpi and assessed by RT-PCR for viral DNA load in brain and lung. (**E**) Brain and lung sections from mice infected with different PRVs (1 × 10^5^ PFU/mice) were analyzed by H&E staining at 3 dpi. The magnified area of each section (right-hand side) is localized by a square on the corresponding lower magnification picture (left-hand side) (scale bar: 100 µm). Data are representative of at least three independent experiments with similar results (mean ± SD of *n* = 3 biological replicates in A and B, *n* = 4 biological replicates in D). *P < 0.05 and **P < 0.01 (two-tailed unpaired t test).

ZBP1, a PRR that orchestrates both IFNs and inflammatory responses, plays a pivotal role during viral infections (21, 22). To determine the role of ZBP1 in PRV infection, we performed viral replication assays on ZBP1-knockdown cell lines (Fig. S4A through C). Both PRV-WT and ΔVP22R exhibited robust replication in control and ZBP1-knockdown cells of the lines SY5Y, 3D4/2, and N2a (Fig. 3A). In contrast, ΔVP22 displayed poor replication in control cells, but ZBP1-knockdown significantly restored higher ΔVP22 titer, by at least 1 log (Fig. 3A). These results were confirmed by using *Zbp1*^+/+^ [wild-type (WT)] and *Zbp1*^−/−^ MEFs isolated from wild-type and *Zbp1*-deficient mice (Fig. 3B; Fig. S4D). Following these results *in vitro*, *Zbp1*^+/+^ (WT) or *Zbp1^−/−^* mice were infected with either PRV-WT, ΔVP22, or ΔVP22R diluted in phosphate-buffered saline (PBS) at 1 × 10^5^ PFU/mice or with the same volume of PBS (mock infected). After infection, survival was monitored. Both WT and *Zbp1^−/−^* mice infected with PRV-WT or ΔVP22R exhibited a 0% survival rate after 7 days. However, distinct outcomes were observed in WT and *Zbp1^−/−^* mice infected with ΔVP22. Among the WT mice, 60% survived infection with ΔVP22, whereas among the *Zbp1^−/−^* mice, none survived (Fig. 3C). At 3 days post-infection (dpi), the increased survival of WT mice infected with ΔVP22 correlated with significantly lower viral loads in brain and lung tissues than those observed in PRV-WT and ΔVP22R-infected WT mice. In *Zbp1*^−/−^ mice infected with the ΔVP22 virus, viral loads in the brain and lungs increased by more than 1 log compared with wild-type mice. In contrast, when *Zbp1*^−/−^ mice were infected with PRV-WT or the VP22-competent virus, the viral loads in the brain and lungs showed only a slight increase (Fig. 3D). Consistently, in WT mice, milder neuropathological lesions in the brain, inflammatory cell infiltrates, and lung congestion were detected in ΔVP22 compared with PRV-WT and ΔVP22R-infected animals, whereas in *Zbp1^−/−^* mice, almost no histological differences were observed between animals infected with VP22-competent or ΔVP22 viruses (Fig. 3E). Taken together, these results suggested that ZBP1 plays a crucial role in controlling PRV infection, which is counteracted by VP22.

### PRV VP22 inhibits ZBP1-mediated inflammatory response

**Fig 4:**
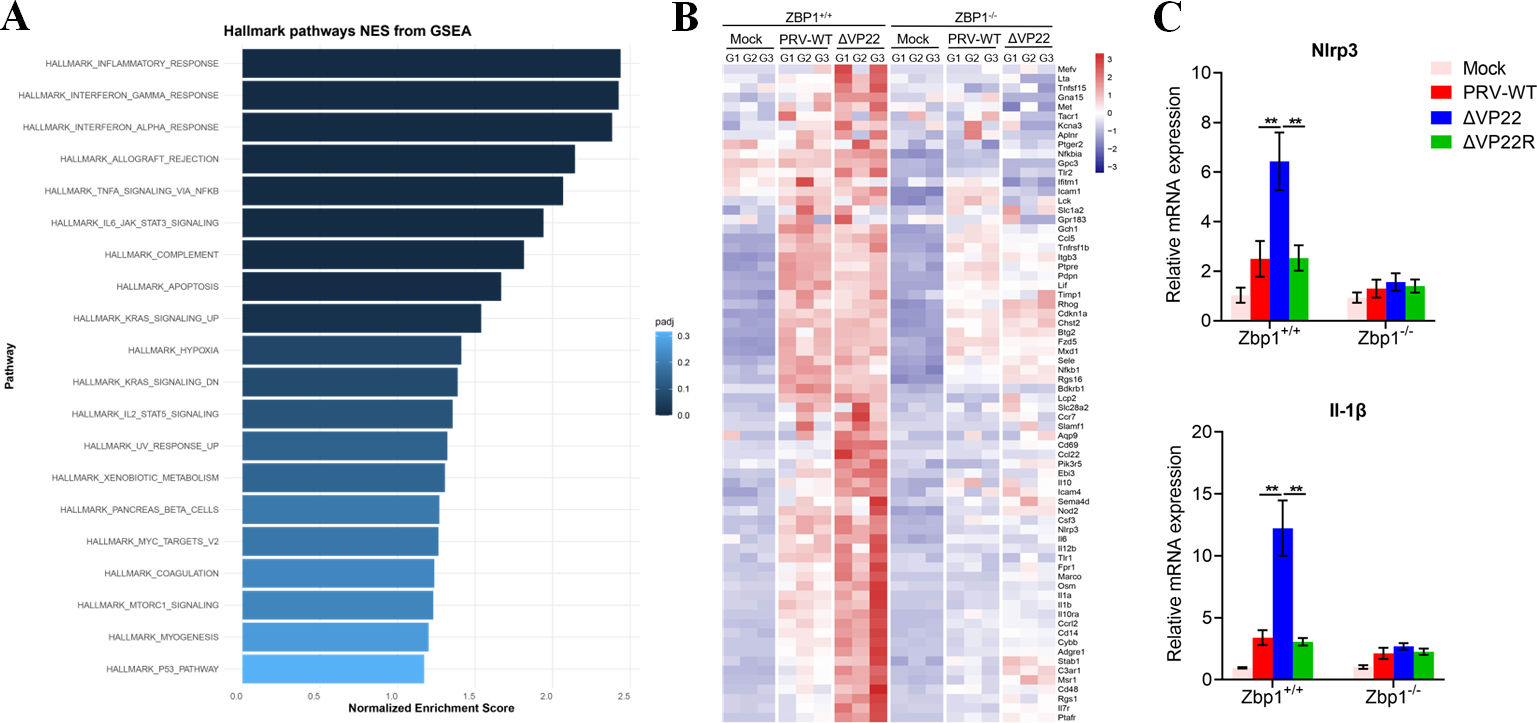
RNA-seq analysis revealed genes differentially expressed during infection with PRV-WT and ΔVP22 in WT or *Zbp1*-deficient BMDMs. (**A**) GSEA hallmark analysis of the upregulated or downregulated genes. (**B**) Heatmap showing the relative expression levels of genes of the inflammatory pathway in mock-infected and PRV-WT or ΔVP22-infected *Zbp1^+/+^* and *Zbp1^−/−^* BMDMs. (**C**) *Zbp1*^+/+^ and *Zbp1^−/−^* BMDMs were infected with PRV-WT, ΔVP22, or ΔVP22R at an MOI of 5; at 12 hpi, the cells were harvested for RNA extraction and RT-PCR quantification of IL-1β and *Nlrp3* mRNAs and 18s rRNA. Data are representative of at least three independent experiments with similar results (mean ± SD of *n* = 3 biological replicates). **P < 0.01 (two-tailed unpaired t test).

To identify the ZBP1-mediated signaling pathway inhibited by VP22, RNA sequencing was performed on *Zbp1*^+/+^ and *Zbp1^−/−^* BMDMs (Fig. S4E) following mock infection or infection with PRV-WT or ΔVP22. The functional pathways associated with the genes differentially expressed in *Zbp1*^+/+^ versus *Zbp1^−/−^* BMDMs, both infected with ΔVP22, were delineated by GSEA analysis. Remarkably, the top enriched pathway was the inflammatory response (Fig. 4A; Fig. S5). Furthermore, heatmap analysis clustered genes involved in inflammatory responses, with different patterns in *Zbp1*^+/+^ and *Zbp1^−/−^* BMDMs (Fig. 4B). In *Zbp1*^+/+^ BMDMs, the expression of inflammatory factors was significantly increased upon infection with ΔVP22 compared with infection with PRV-WT. However, in *Zbp1^−/−^* BMDMs, the differences between ΔVP22 and PRV-WT were abolished. Among the genes of interest, i.e., which were upregulated in the absence of VP22 in *Zbp1^+/+^* BMDMs and depended on functional *Zbp1*, *Nlrp3* and *Il-1β* are the most prominent genes related to the inflammatory response. Their expression patterns were confirmed by qPCR (Fig. 4C). Collectively, this analysis suggested that PRV VP22 interfered with ZBP1-mediated inflammasome response triggered by PRV.

### PRV VP22 interacts with ZBP1

**Fig 5:**
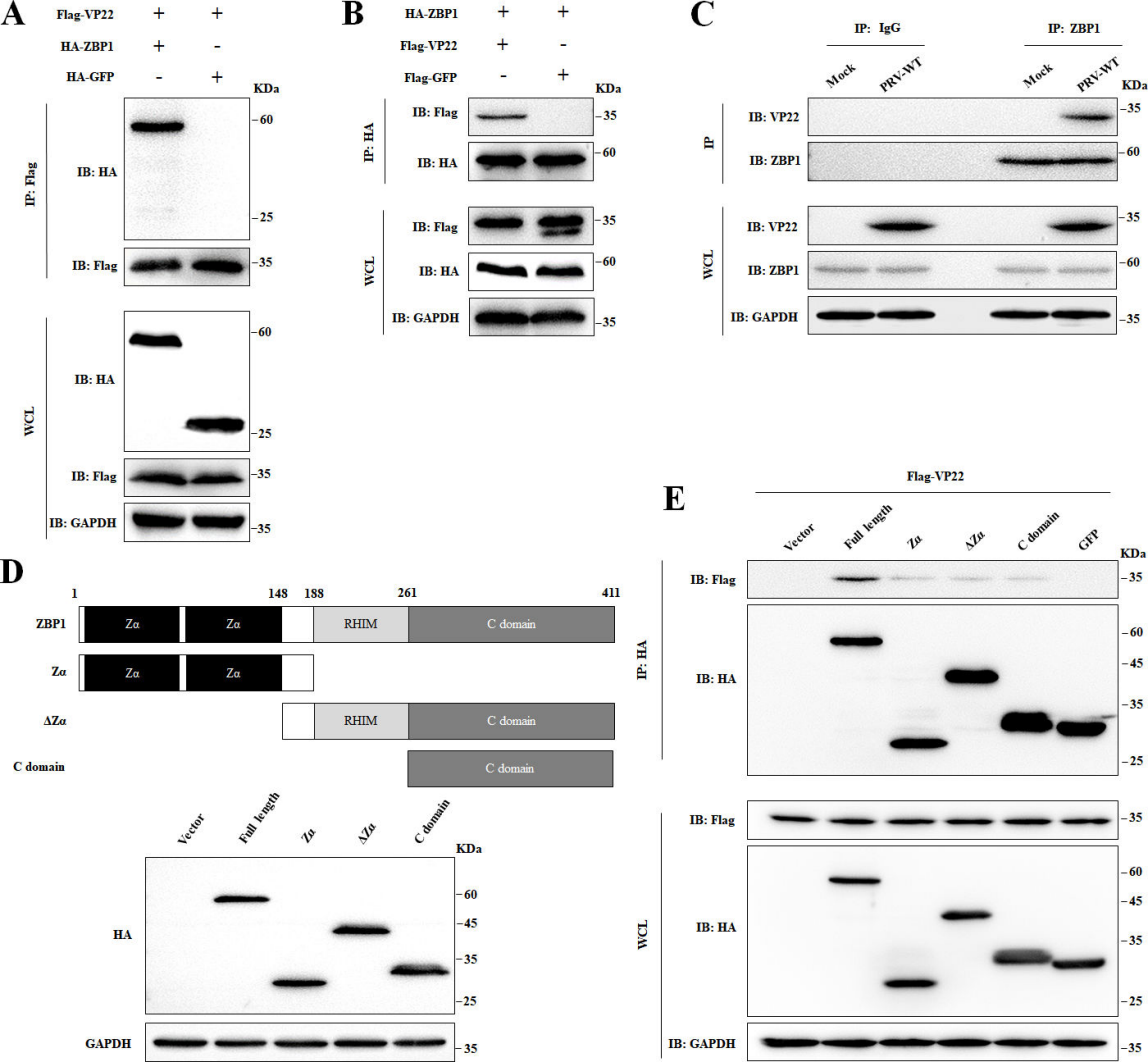
PRV VP22 directly binds ZBP1. (**A**) HEK-293 T cells were transfected with 2 µg of a plasmid driving the expression of Flag-VP22, together with 2 µg of a plasmid driving either the expression of HA-ZBP1 or HA-GFP. At 24 hours post-transfection (hpt), cells were processed for IP with anti-Flag magnetic beads. Whole-cell lysates (WCLs) and precipitated proteins were analyzed by western blots probed with antibodies against Flag-tag, HA-tag, and GAPDH. (**B**) HEK-293 T cells were transfected with 2 µg of a plasmid driving the expression of HA-ZBP1, together with 2 µg of a plasmid driving either the expression of Flag-VP22 or that of Flag-GFP. At 24 hpt, cells were processed for IP with anti-HA magnetic beads. WCLs and precipitated proteins were analyzed by western blots probed with antibodies against Flag-tag, HA-tag, and GAPDH. (**C**) MEFs were mock infected or PRV infected with an MOI of 5. After 24 hours of incubation, cell lysates were prepared and immunoprecipitated with anti-ZBP1 antibody or control rabbit IgG. The level of coprecipitated VP22, as well as ZBP1 and VP22, in whole cell lysates was assessed by immunoblotting with anti-VP22, anti-ZBP1, and anti-GAPDH antibodies. (**D**) Schematic representation of WT and truncated ZBP1. HEK-293 T cells were transfected with 2 µg of either a HA-empty vector or a vector encoding HA-ZBP1 or one of its truncated mutants (HA-ZBP1-Zα, HA-ZBP1-ΔZα, or HA-ZBP1-C domain). At 24 hpt, cells were harvested and analyzed by western blot for protein production with antibodies against HA-tag and GAPDH. (**E**) HEK-293 T cells were transfected with 2 µg of a vector promoting the production of Flag-VP22 together with 2 µg of either an empty HA vector or a vector promoting the production of HA-ZBP1, HA-ZBP1-Zα, HA-ZBP1-ΔZα, HA-ZBP1-C domain, or HA-GFP. At 24 hpt, cells were processed for IP with anti-HA magnetic beads. WCLs and precipitated proteins were quantified by western blots using antibodies against HA-tag, Flag-tag, and GAPDH. All the data are representative of at least three independent experiments with similar results.

Next, we investigated the mechanism by which VP22 could counteract ZBP1 function. The interaction between ZBP1 and VP22 was first probed by immunoprecipitation (IP) assay using ZBP1 and VP22 recombinant proteins, respectively, tagged with Human influenza hemagglutinin (HA) and Flag epitopes. As anticipated, the protein HA-ZBP1, but not the irrelevant control HA-tagged Green Fluorescent Protein (HA-GFP), precipitated with Flag-VP22 via an anti-Flag antibody (Fig. 5A). Reciprocally, VP22 specifically precipitated with HA-ZBP1 using an anti-HA antibody (Fig. 5B). Moreover, we demonstrated that VP22 co-precipitated with endogenous ZBP1 in virus-infected cells (Fig. 5C). Additionally, we sought to delineate the functional domain of ZBP1 that interacted with VP22. To achieve this, we constructed the truncated ZBP1 proteins Zα, ΔZα, and C-domain, all of which were expressed at adequate levels (Fig. 5D). Interestingly, the three truncated proteins Zα, ΔZα, and C-domain promoted little co-precipitation of VP22 in IP assay; only full-length ZBP1 yielded high co-precipitated VP22 (Fig. 5E), underscoring the necessity of full-length ZBP1 for VP22 binding.

### PRV VP22 inhibits ZBP1-mediated NLRP3 inflammasome activation

**Fig 6:**
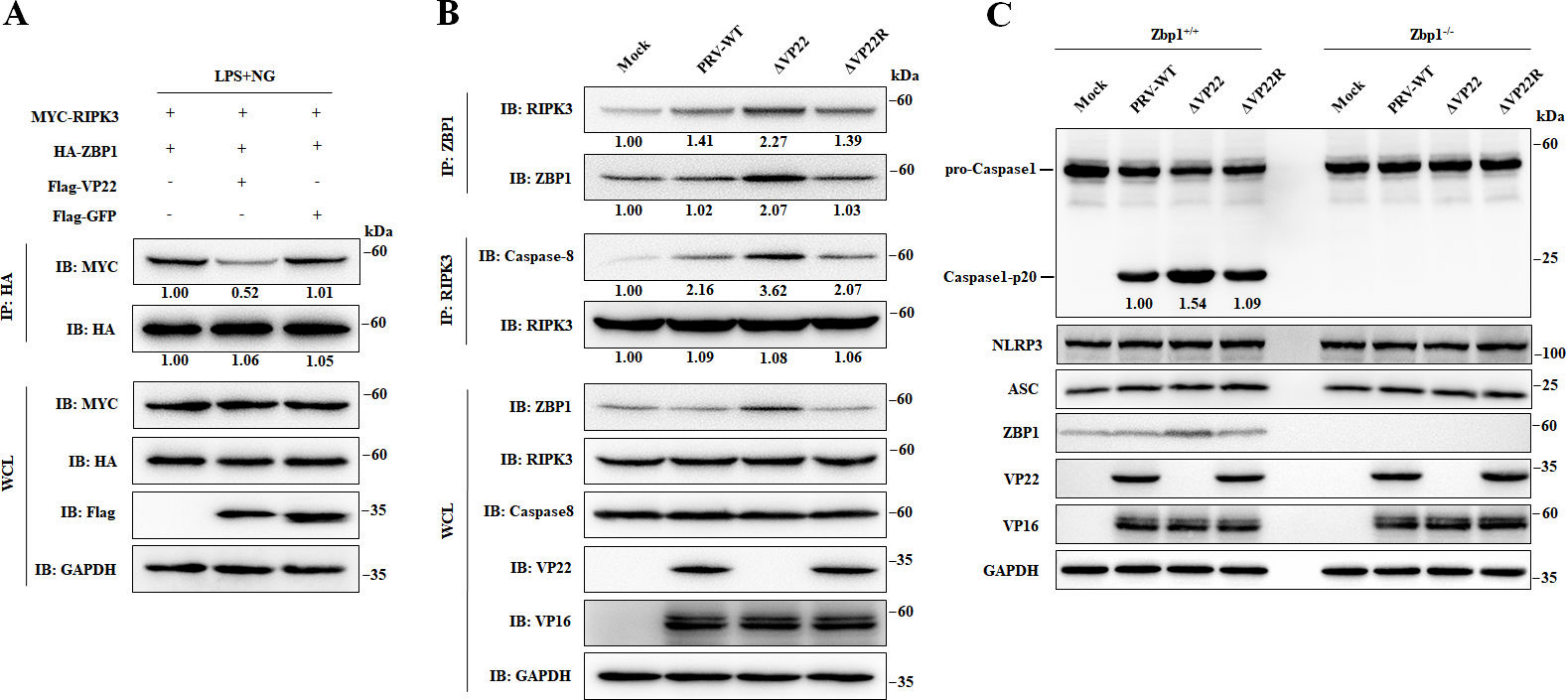
PRV VP22 inhibits ZBP1-mediated inflammatory responses. (**A**) THP-1 cells were transfected with 2 µg of a plasmid promoting the expression of MYC-tagged RIPK3 (MYC-RIPK3) and 2 µg of a plasmid promoting the expression of HA-ZBP1, together with 2 µg of either an empty vector, a plasmid driving either the expression of Flag-VP22, or that of Flag-GFP, and then stimulated with Lipopolysaccharide (LPS, 1 µg/mL) plus Nigericin (NG, 2 µM). At 24 hpt, cells were processed for IP with anti-HA magnetic beads. WCLs and precipitated proteins were analyzed by western blots with antibodies against MYC-tag, HA-tag, Flag-tag, and GAPDH. (**B**) BMDMs were mock infected or infected with PRV-WT, ΔVP22, or ΔVP22R at 5 PFU/cell. At 12 hpi, cells were processed for IP with anti-ZBP1 or anti-RIPK3. WCLs and precipitated proteins were analyzed by western blots with antibodies against ZBP1, RIPK3, Caspase-8, VP16, VP22, and GAPDH. (**C**) *Zbp1^+/+^* and *Zbp1^−/−^* BMDM cells were mock infected or infected with PRV-WT, ΔVP22, or ΔVP22R at 5 PFU/cell. At 12 hpi, cells were harvested for western blot analysis with antibodies against ZBP1, VP22, NLRP3, ASC, Caspase-1, VP16, and GAPDH. The intensities of the western blot bands were analyzed using ImageJ. The vector or mock group was set as 1.00. All the data are representative of at least three independent experiments with similar results.

Emerging evidence suggests that ZBP1 activates the NLRP3 inflammasome during certain viral infections, leading to IL-1β production and inflammation (23–25). Since we found that VP22 could bind ZBP1, we asked whether VP22 would disrupt the interaction between ZBP1 and NLRP3 or compete with NLRP3 for binding to ZBP1. Previous studies have shown that ZBP1 activates the NLRP3 inflammasome by recruiting RIPK3 (26). Therefore, we speculated that VP22 might inhibit ZBP1/RIPK3 binding, thereby preventing the formation of the NLRP3 inflammasome. To explore this hypothesis, VP22 was tested for its ability to prevent ZBP1/RIPK3 interaction in IP assays. This experiment indicated that VP22 significantly inhibited the binding of ZBP1 to RIPK3 (Fig. 6A).

RIPK3 further binds Caspase-8 to activate downstream NLRP3, promoting the formation of the NLRP3 inflammasome (27). Therefore, we examined the interactions between RIPK3 and ZBP1 or Caspase-8 during viral infections (Fig. 6B). ZBP1/RIPK3 and RIPK3/Caspase-8 interactions were stronger in cells infected with VP22-null virus (ΔVP22) compared with PRV-WT or VP22-repaired (ΔVP22R) viruses. NLRP3 inflammasome is a trimeric complex composed of NLRP3, ASC, and Caspase-1. Upon activation, NLRP3 interacts with ASC, which further recruits and activates Caspase-1, thereby inducing a pro-inflammatory response. Thus, we examined the expression of proteins related to the NLRP3 inflammasome in *Zbp1^+/+^* and *Zbp1^−/−^* BMDMs. As expected, in *Zbp1^+/+^* BMDMs, Caspase-1 was mildly activated following infection with PRV-WT and PRV-ΔVP22R but strongly activated after infection with PRV-ΔVP22. However, this response was abolished in *Zbp1^−/−^* BMDMs (Fig. 6C). In summary, this set of experiments suggests that PRV VP22 hinders the recruitment of RIPK3 by ZBP1, impeding RIPK3/Caspase-8 binding, which ultimately suppresses the activation of the NLRP3 inflammasome.

### The N-terminal 1–50 aa domain of PRV VP22 inhibits ZBP1-mediated NLRP3 inflammasome activation

**Fig 7:**
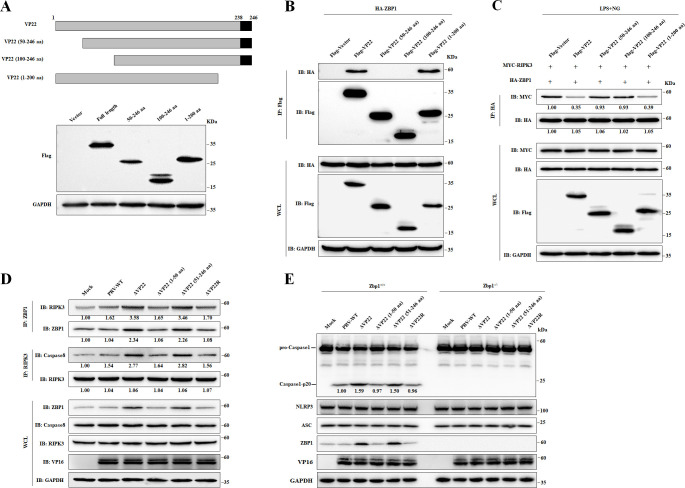
The N-terminal 1–50 aa domain of PRV VP22 is responsible for inhibiting ZBP1-mediated inflammatory responses. (**A**) Schematic representation of WT and truncated recombinant ZBP1 proteins. HEK-293 T cells were transfected with 2 µg of either Flag-empty vector, Flag-VP22, or one of its truncated mutants [Flag-VP22 (50–246 aa), Flag-VP22 (100–246 aa), or Flag-VP22 (1–200 aa)]. At 24 hpt, cells were harvested for western blot analysis with antibodies against Flag-tag and GAPDH. (**B**) HEK-293 T cells were transfected with 2 µg of a vector promoting the expression of HA-ZBP1 together with 2 µg of either a Flag-empty vector or a plasmid encoding Flag-VP22 (full length), Flag-VP22 (50–246 aa), Flag-VP22 (100–246 aa), or Flag-VP22 (1–200 aa). At 24 hpt, cells were processed for IP with anti-Flag magnetic beads. WCLs and precipitated proteins were analyzed by western blots with antibodies against Flag-tag, HA-tag, and GAPDH. (**C**) THP-1 cells were transfected with 2 µg of a plasmid encoding MYC-RIPK3 and 2 µg of a plasmid encoding HA-ZBP1, together with 2 µg of either a Flag-empty vector or a plasmid encoding Flag-VP22 (full length), Flag-VP22 (50–246 aa), Flag-VP22 (100–246 aa), or Flag-VP22 (1–200 aa) and then stimulated with LPS (1 µg/mL) plus NG (2 µM). At 24 hpt, cells were processed for IP with anti-HA magnetic beads. WCLs and precipitated proteins were analyzed by western blot with antibodies against MYC-tag, HA-tag, Flag-tag, and GAPDH. (**D**) BMDMs were mock infected or infected with PRV-WT, ΔVP22, or ΔVP22R at 5 PFU/cell. At 12 hpi, cells were processed for IP with anti-ZBP1 or anti-RIPK3. WCLs and precipitated proteins were analyzed by western blots with antibodies against ZBP1, RIPK3, Caspase-8, VP16, and GAPDH. (**E**) *Zbp1^+/+^* and *Zbp1^−/−^* BMDMs were mock infected or infected with PRV-WT, ΔVP22, ΔVP22 (1–50 aa), ΔVP22 (51–246 aa), or ΔVP22R at 5 PFU/cell. At 12 hpi, cells were harvested for western blot analysis with antibodies against ZBP1, NLRP3, ASC, Caspase-1, VP16, and GAPDH. The intensities of the western blot bands were analyzed using ImageJ. The vector or mock group was set as 1.00. All the data are representative of at least three independent experiments with similar results.

To identify the domain of VP22 that inhibited ZBP1-mediated NLRP3 inflammasome activation, three truncated VP22 recombinant proteins were constructed (Fig. 7A). IP assays showed that the N-terminal 1–50 Amino acid (aa) domain of VP22 was essential for its interaction with ZBP1 (Fig. 7B). Consistently, the 1–50 aa domain of VP22 significantly inhibited the binding of ZBP1 to RIPK3 (Fig. 7C). To further demonstrate the inhibitory role of the 1–50 aa domain of VP22 on ZBP1-mediated activation of NLRP3 inflammasome, recombinant viruses carrying deletions of aa 1–50, named ΔVP22 (51–246 aa), or aa 51–246, named ΔVP22 (1–50 aa), were constructed (Fig. S6). As anticipated, the interaction between RIPK3 and ZBP1 was stronger following ΔVP22 or ΔVP22 (51–246 aa) infections than following infection with viruses with intact VP22 1–50 aa domain. Similar results were observed for RIPK3 and Caspase-8 interaction (Fig. 7D). Consistently, in *Zbp1^+/+^* BMDMs, Caspase-1 was mildly activated following infection with PRV-WT, ΔVP22 (1–50 aa), or ΔVP22R but strongly activated upon infection with PRV-ΔVP22 and ΔVP22 (51–246 aa). This pattern was abolished in *Zbp1^−/−^* BMDMs, supporting that ZBP1 plays a central role in PRV-induced inflammasome response (Fig. 7E). Altogether, these results demonstrated that the N-terminal 1–50 aa domain of VP22 is responsible for inhibiting ZBP1-mediated NLRP3 inflammasome activation during PRV infection.

### The N-terminal 1–50 aa domain of PRV VP22 contributes to viral infection

**Fig 8:**
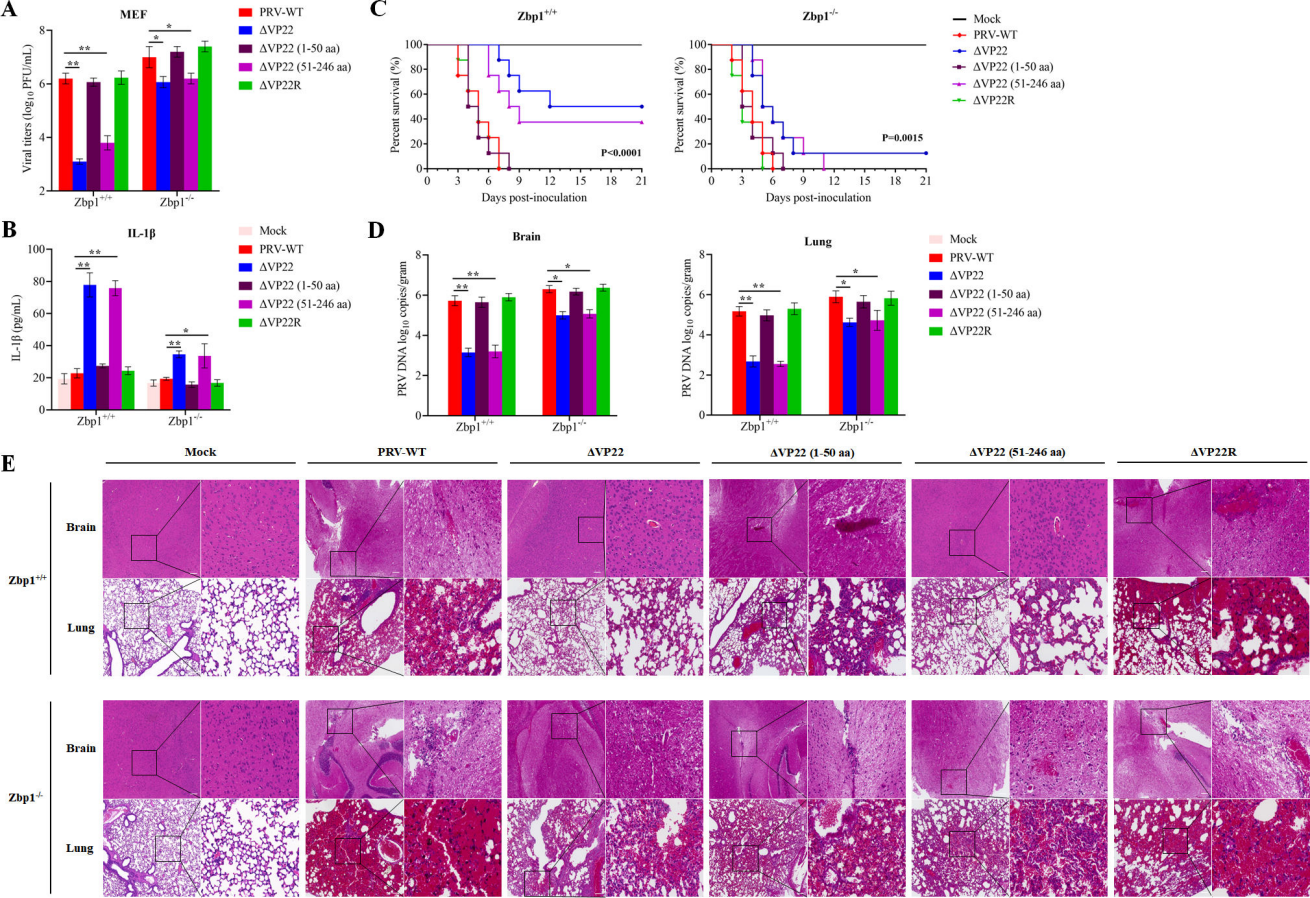
ZBP1 is essential for controlling PRV infection. (**A**) *Zbp1*^+/+^ and *Zbp1*^−/−^ MEFs were mock infected or infected with PRV-WT, ΔVP22, ΔVP22 (1–50 aa), ΔVP22 (51–246 aa), or ΔVP22R at 0.01 PFU/cell. At 48 hpi, cells and supernatants were harvested, and the total virus titers were determined by a plaque assay in Vero cells. (**B**) *Zbp1*^+/+^ and *Zbp1^−/−^* BMDMs were infected with PRV-WT, ΔVP22, or ΔVP22R at an MOI of 5. At 12 hpi, the cell supernatants were harvested for IL-1β quantification by enzyme-linked immunosorbent assay (ELISA). (**C**) *Zbp1*^+/+^ and *Zbp1*^−/−^ mice separately housed in six groups (*n* = 8 mice/group) were mock infected or infected with either PRV-WT, ΔVP22, ΔVP22 (1–50 aa), ΔVP22 (51–246 aa), or ΔVP22R (1 × 10^5^ PFU/mice) by intraperitoneal injection and monitored for survival. Simple survival analyses for the six groups using Kaplan–Meier curves (*Zbp1*^+/+^: *P* < 0.0001; *Zbp1*^−/−^: *P* = 0.0015). (**D**) Viral DNA loads in the brain, lung, or brainstem of mice infected as in **C** were quantified by RT-PCR at 3 dpi (*n* = 4 mice/group). (**E**) Brain and lung sections from mice infected as in **C** and **D** were analyzed by H&E staining at 3 dpi. The magnified area of each section (right-hand side) is localized by a square on the corresponding lower-magnification picture (left-hand side) (scale bar: 100 µm). Data are representative of at least three independent experiments with similar results (mean ± SD of *n* = 3 biological replicates in A and B, *n* = 8 biological replicates in C, *n* = 4 biological replicates in D). *P < 0.05 and **P < 0.01 (two-tailed unpaired t test).

Last, the significance of the N-terminal 1–50 aa domain of PRV VP22 for viral infection was evaluated. The replication of ΔVP22 and ΔVP22 (51–246 aa) in *Zbp1^−/−^* MEFs increased by at least 2 logs compared with that in *Zbp1*^+/+^ MEFs. However, only slight changes between *Zbp1^+/+^* and *Zbp1^−/−^* MEFs were observed for viruses containing an intact N-terminal 1–50 aa VP22 domain (Fig. 8A). To assess the relationship between VP22 and IL-1β induction, *Zbp1*^+/+^ or *Zbp1^−/−^* BMDMs were either mock infected or infected with different PRVs. In *Zbp1*^+/+^ BMDMs, the IL-1β level was significantly higher upon infection with ΔVP22 or ΔVP22 (51–246 aa) than with viruses containing an intact N-terminal 1–50 aa VP22 domain. In contrast, in *Zbp1^−/−^* BMDMs, these differences in IL-1β induction between the various viruses were significantly reduced (Fig. 8B). Next, *Zbp1^+/+^* or *Zbp1^−/−^* mice were mock infected (PBS) or infected with different viruses at 1 × 10^5^ PFU/mouse and monitored for survival. Different survival patterns were observed for the *Zbp1^+/+^* and *Zbp1^−/−^* mice infected with ΔVP22 or ΔVP22 (51–246 aa) (Fig. 8C). Among the *Zbp1^+/+^* mice, 4/8 and 3/8 survived after infection with ΔVP22 and ΔVP22 (51–246 aa), respectively. In contrast, among the *Zbp1*^−/−^ mice, only 1/8 and 0/8 survived after infection with ΔVP22 and ΔVP22 (51–246 aa), respectively. The brain and lung tissues of mice treated identically were collected at 3 dpi to determine PRV DNA loads. In *Zbp1^+/+^* mice, PRV loads in lung and brain were significantly lower with ΔVP22 or ΔVP22 (51–246 aa) than with PRV-WT, ΔVP22 (1–50 aa), or ΔVP22R PRVs. These differences in viral loads between viruses containing or not an intact N-terminal 1–50 aa VP22 domain were reduced in *Zbp1*^−/−^ mice (Fig. 8D). In keeping with these results, histological analysis indicated milder brain and lung lesions in *Zbp1^+/+^* mice infected with ΔVP22 or ΔVP22 (51–246 aa) than in those infected with PRV-WT, ΔVP22 (1–50 aa), or ΔVP22R. In contrast, in *Zbp1^−/−^* mice infected with ΔVP22 or ΔVP22 (51–246 aa), the lesions were similar to those in mice infected with PRV-WT, ΔVP22 (1–50 aa), or ΔVP22R (Fig. 8E). Altogether, these experiments showed that the N-terminal 1–50 aa domain of PRV VP22 contributes to viral infection.

## DISCUSSION

VP22 homologous protein, encoded by *UL49*, is a key component of the herpesviruses (28). In HSV-1, VP22 is known to antagonize cGAS-STING signaling, inhibit AIM2-dependent inflammasome activation, and suppress CD1d-mediated antigen presentation (29–31). However, the role of VP22 in PRV remains largely unexplored. Herein, we demonstrate that PRV VP22 contributes to viral replication and pathogenicity. Mechanistically, PRV VP22 interferes with ZBP1-mediated NLRP3 inflammasome. This study deepens our understanding of the pathogenicity of PRV and other alpha-herpesviruses.

As a potent innate immune sensor, ZBP1 plays an essential role against multiple pathogen infections, particularly viruses. ZBP1 detects invasive virus-derived nucleic acids and disrupts the viral replication niche by initiating cell death. During evolution, viruses have evolved various strategies to block ZBP1 signaling and ensure efficient infection. During IAV infection, ZBP1-mediated PANoptosis significantly suppresses viral replication. However, the inflammatory death of pulmonary cells also leads to lethality in infected mice due to pulmonary dysfunction and inflammation (8–10, 32). During flavivirus infections, ZBP1 acts as a protective factor and is significantly increased in the brain of mice infected with Zika Virus or West Nile Virus (WNV) (33, 34). The absence of ZBP1 results in higher viral loads in the brain, more severe viremia, and significantly increased morbidity and mortality in WNV-infected mice (34). In this study, we demonstrate that PRV VP22 functions as a virus-encoded virulence factor by evading the inhibitory effects of ZBP1. Mechanistically, PRV VP22 interacts with ZBP1, impeding the recruitment of RIPK3 and Caspase-8, thereby inhibiting the activation of NLRP3 inflammasome. Nevertheless, an important question remains to be elucidated: how is ZBP1 activated following VP22-null PRV infection? And specifically, does ZBP1 sense Z-DNA or Z-RNA?

ZBP1 possesses two tandem Z-nucleic acid (Z-NA)-binding domains (Zα1 and Zα2) at its N-terminus, followed by two receptor-interacting protein (RIP) homotypic interaction motifs (RHIMs), and a C-terminal signal domain (SiDo) (35). The Zα domains specifically bind Z-DNA and Z-RNA, with the Zα2 domain acting as a molecular switch to trigger PANoptosis during viral infections (23, 36). The RHIMs facilitate the recruitment of RHIM-containing kinases, such as receptor-interacting serine/threonine kinase 1 (RIPK1) and RIPK3, leading to NF-κB activation, inflammation, and context-dependent programmed cell death (37). The C-terminal SiDo induces IFN-I responses by interacting with TANK-binding kinase 1 and IRF3 in response to immunostimulatory DNA (35). We have demonstrated that the Zα domains, RHIMs, and C-terminal SiDo are necessary for binding VP22, but the significance of this interaction requires further study. Notably, several studies have demonstrated that ZBP1 expression is upregulated upon viral infections, including SARS-CoV-2, IAV, and HSV-1 (5, 10, 12). In our study, we found that ZBP1 mRNA levels were upregulated following PRV infection. Regarding ZBP1 protein levels, we observed an upregulation only in the presence of the VP22 deletion virus. This suggests that VP22 may reduce ZBP1 expression at the protein level, a phenomenon that warrants further investigation.

Inflammatory responses are critical for controlling and clearing PRV infection (19, 38, 39). It has been suggested that PRV infection triggers AIM2 inflammasome (39). It is noteworthy that, in HSV-1, VP22 interacts with AIM2 and prevents its oligomerization, thereby inhibiting the activation of AIM2-dependent inflammasome. This suppression reduces the secretion of IL-1β and IL-18, which favors viral replication (31). ZBP1 is also a crucial regulator of the NLRP3 inflammasome in response to viral infection. Interestingly, we found that the VP22 homologous protein in PRV hinders ZBP1-mediated NLRP3 inflammasome activation. PRV VP22 disrupts the binding between ZBP1 and RIPK3/Caspase-8 and inhibits the cleavage of Caspase-1 and IL-1β induction, thereby promoting viral infection. However, whether this weakened interaction is due to VP22 inhibiting ZBP1 expression, thereby affecting ZBP1-induced inflammasome formation, still requires further investigation. Furthermore, during HSV-1 infection, AIM2 has been shown to regulate the innate immune sensors ZBP1 and pyrin. This interaction modulates their activities, driving inflammatory signaling and a form of inflammatory cell death known as PANoptosis, thereby enhancing the host’s immune defense against the virus (12). More and more studies have shown that AIM2 and ZBP1 are crucial intracellular immune sensors, essential for the body to recognize different types of pathogens and danger signals, thereby triggering inflammatory responses (40–42). Furthermore, they are closely associated with the formation of a new form of PANoptosis. Therefore, it will be essential to investigate whether the AIM2 or AIM2-ZBP1 axis is regulated by PRV VP22 in future studies.

In summary, this study demonstrates the crucial role of ZBP1 in pro-inflammatory cytokine release during PRV infection. ZBP1 not only resists PRV replication but also plays a vital role in host defense against PRV. The ZBP1-VP22 axis offers new insights for the prevention and control of PRV infection.

## MATERIALS AND METHODS

### Cells, viruses, and plasmids

Human embryonic kidney (HEK)-293T (CRL-11268), Vero (CCL-81), Neuro-2a (N2a; CCL-131), SH-SY5Y (CRL-2266), 3D4/2 (CRL-2845), and THP-1 (TIB-202) cell lines were obtained from the American Type Culture Collection. WT MEFs or mouse BMDMs and ZBP1^−/−^ MEFs or BMDMs were isolated from C57BL/6 wild-type and ZBP1^−/−^ knockout mouse embryos, respectively. ZBP1-knockdown cell lines were generated by using the shRNA lentiviral system, with puromycin at a concentration of 5 µg/mL for selection (Selleck, #S9631). All cells were grown in Dulbecco’s modified Eagle’s medium (DMEM; Gibco, USA) containing 10% fetal bovine serum (FBS; Gibco, USA) in a humidified 5% CO_2_ incubator at 37°C. PRV ZJ01 (GenBank: KM061380.1) is a prototype PRV strain used in this study as PRV-WT. The recombinant viruses with *VP22* gene deletion (ΔVP22), its revertant (ΔVP22R), or carrying mutations in VP22-specific domains, i.e., depleted for aa 1–50, named ΔVP22 (51–256 aa) or for aa 51–246, named as ΔVP22 (1–50 aa), were generated by the CRISPR-Cas9 system as described below. Viral stock preparation and infectivity titrations were conducted in Vero cells. The plasmid encoding Flag-VP22 was obtained by inserting the PRV *UL49* gene between the *Bam*H I and *Xho* I sites of pCMV-N-Flag (Beyotime, #D2722). Flag-VP22-encoding cDNA was used as a PCR template to generate truncated VP22 mutants, specifically Flag-VP22 (1–200 aa), Flag-VP22 (50–246 aa), and Flag-VP22 (100–246 aa), which were subsequently inserted between the *Bam*H I and *Xho* I sites of pCMV-N-Flag. The GFP cassette was inserted into pCMV-N-HA (Clontech, #635690) or pCMV-N-Flag by using the *Eco*R I and *Xho* I sites to generate HA-GFP and Flag-GFP, respectively. pX335 (#42335), pLKO.1 puro (#8453), and lentivirus package plasmids pMD2.G (#12259) and psPAX2 (#12260) were purchased from Addgene. HA-ZBP1 was obtained by inserting mouse *Zbp1* cDNA between the *Bam*H I and *Xho* I sites of pCMV-N-HA. HA-ZBP1 was used as a PCR template to generate cDNA encoding the truncated ZBP1 mutants HA-ZBP1-Zα (encompassing aa 1–188), HA-ZBP1-ΔZα (encompassing aa 148–411), and HA-ZBP1-C domain (encompassing aa 261–411), individually inserted between the *Bam*H I and *Xho* I sites of pCMV-HA. MYC-RIPK3 was obtained by inserting mouse *Ripk3* cDNA between the *Hin*d III and *Xho* I sites of pCMV-N-MYC. The primers used for plasmid construction are listed in Table S1.

### Antibodies

The antibodies and chemical reagents used in this study are as follows: anti-FLAG (HRP conjugate, M2; Sigma-Aldrich, #A8592), anti-HA (HRP conjugate, 6E2; Cell Signaling Technology, #2999), anti-GAPDH (Proteintech, #60004-1-Ig), anti-ZBP1 (Cell Signaling Technology, #33402; Santa Cruz Biotechnology, sc-271483), anti-RIPK3 (Cell Signaling Technology, #95702; Santa Cruz Biotechnology, sc-374639), anti-Caspase-8 (Cell Signaling Technology, #4790; Santa Cruz Biotechnology, sc-81656), anti-Caspase-1 (AdipoGen, AG-20B-0042), anti-NLRP3 (AdipoGen, AG-20B-0014-C100), anti-ASC (Santa Cruz Biotechnology, sc-514414), mouse anti-rabbit IgG-HRP (Santa Cruz Biotechnology, sc-2357), and goat anti-mouse IgG-HRP (Santa Cruz Biotechnology, sc-2005). The anti-VP22 and anti-VP16 antibodies were prepared in-house; anti-unique long region 54 (UL54) was a gift from Dr. Chunfu Zheng, University of Calgary, Canada (43).

### Mice

WT and *Zbp1*^−/−^ mice and were purchased from the Nanjing Biomedical Research Institute of Nanjing University. All mice were housed in specific pathogen-free barrier facilities in Nanjing Agricultural University. MEFs were isolated from 15-day post-coitum embryos obtained from euthanized pregnant mice. The isolated cells were plated in cell culture flask and then cultured in DMEM containing 10% FBS. For the generation of BMDMs, bone marrow cells were isolated from the tibia and femur of 6–8-week-old *Zbp1*^−/−^ or WT mice and plated in dishes at a density of 1 × 10^6^ cells/mL. The cells were then cultured in DMEM containing 20% FBS and 30% L929 supernatant for 4 days, after which the medium was replaced. The cells were cultured for another 4 days, and the BMDMs were harvested as the adherent cells, which were then infected with PRV. *In vivo* PRV challenges were performed in 6-week-old WT and *Zbp1^−/−^* knockout mice of similar weight.

### Transfection

HEK-293T cells were transfected with different plasmids using Lipofectamine 3000 (Thermo Fisher Scientific). THP-1 cells were transfected with different plasmids using Lipofectamine LTX & Plus Reagent (Thermo Fisher Scientific) together with NATE (InvivoGen). All transfections followed the manufacturer’s protocols.

### Generation of recombinant PRV by the CRISPR-Cas9 system

To construct the plasmids pX335-*VP22* singular guide RNA (sgRNA) 1 and pX335-*VP22* sgRNA2, the sgRNA1 and sgRNA2 sequences targeting PRV *VP22* were obtained by fusing sgRNA1-Fwd and sgRNA1-Rev oligonucleotides, respectively, and sgRNA2-Fwd and sgRNA2-Rev oligonucleotides, respectively. These fragments were then inserted into the pX335 vector at the *Bbs* I locus to generate the recombinant plasmids pX335-*VP22* sgRNA1 and pX335-*VP22* sgRNA2, respectively. The plasmids pX335-*GFP* sgRNA1 and pX335-*GFP* sgRNA2 were obtained as previously described (44). To generate the donor plasmids pUC19-*UL50-GFP-UL48*, pUC19-*UL50-UL48*, pUC19-*UL50-VP22(UL49)-UL48*, pUC19-*UL50-VP22 (1–50 aa)-UL48*, and pUC19-*UL50-VP22 (51–246 aa)-UL48*, we integrated the corresponding DNA fragments into pUC19 using the *Hin*d III, *Pst* I, *Kpn* I, and *Eco*R I restriction sites. All oligonucleotide sequences are detailed in Table S1. All recombinant plasmids were verified by Sanger sequencing. Initially, recombinant viruses lacking *VP22* but containing *GFP*, designated as ΔVP22 (GFP), were generated by transfecting the donor plasmid pUC19-*UL48-GFP-UL50* along with pX335-*VP22* sgRNA1 and pX335-*VP22* sgRNA2 into HEK-293T cells. At 24 hours post-transfection, the cells were infected with PRV-WT at a multiplicity of infection (MOI; number of viral particles/number of cells) of 0.1. By 48 hours post-infection, cell supernatants were collected for recombinant virus isolation. The supernatants were serially diluted and inoculated to Vero cells. Viral plaques were selected based on GFP fluorescence. Following five rounds of purification, the virus was verified by PCR, western blot, and sequence analyses. This initial virus was named ΔVP22 (GFP) and used as precursor to derive ΔVP22, ΔVP22R, ΔVP22 (1–50 aa), and ΔVP22 (51–246 aa) according to the same procedures as for ΔVP22 (GFP) construction, but selecting for plaques lacking GFP fluorescence.

### Generation of *Zbp1* knockdown cell lines by lentiviral transduction

pLKO.1 puro *Zbp1* Target shRNA Plasmid and pLKO.1 puro Non-Target shRNA Control Plasmid (negative control) were engineered by ligating shRNAs into pLKO.1 using *Age* I and *Eco*R I digestion. The resulting plasmids, together with pMD2.G and psPAX2 plasmids, were transfected into HEK-293 T cells at equimolar proportion. At 48 hours post-transfection, lentiviral particles were harvested following a freeze-thaw cycle and filtered through a 0.22-µm membrane. Filtered lentivirus was utilized to infect SH-SY5Y, N2a, and 3D4/2 cell lines. At 48 hours post-infection, the cells were overlaid with fresh medium containing 5 µg/mL puromycin. All experiments were performed within 2 weeks following lentiviral transduction.

### RNA sequencing and data analysis

Total RNA from mock or virus-infected WT and *Zbp1*^−/−^ MEFs was extracted using the RNeasy Plus Mini Kit (Qiagen, #74104) and subjected to RNA-deep sequencing (RNA-seq) analysis (Novogene). Sequencing libraries were generated with the NEBNext Ultra RNA Library Prep Kit for Illumina following the manufacturer’s instructions, with index codes added to assign the sequences to their respective sample origin. Clustering of the index-coded samples was performed on a cBot Cluster Generation System using TruSeq PE Cluster Kit v3-cBot-HS (Illumina). After cluster generation, the prepared libraries were sequenced on an Illumina HiSeq platform, generating 125-bp/150-bp paired-end reads. All analyses were conducted according to methods previously described (45).

### qPCR analysis

Total RNA was harvested from cells by using an RNeasy Plus Mini Kit (Qiagen, #74104) according to the manufacturer’s instruction. Aliquots of RNA (about 200 ng) were used for cDNA synthesis with a HiScript II Q RT SuperMix for qPCR (Vazyme, #R223-01). The qPCRs were performed on an Applied Biosystems ABI Prism 7900 H T instrument with an AceQ qPCR SYBR Green Master Mix (Vazyme, #Q111-02). For the detection of genes in transcriptomics, the gene expression levels were normalized to that of endogenous control 18S rRNA. Relative gene expression was determined as described previously (45). Primer sequences used for the detection of mouse *Ddx58*, *Dhx58*, *Ifih1*, *Tlr3*, *Zbp1*, *Nlrp3*, *Il-1β*, and *Il-1α* mRNAs, and 18S rRNA are listed in Table S1. The viral loads in cell, brain, and lung samples from PRV-infected mice were determined by absolute quantification of gB (PRV glycoprotein B) gene by RT-PCR, following previously described methods (46), with the primers listed in Table S1.

### Immunoprecipitation assays

For immunoprecipitation, cellular substrates were harvested and lysed using RIPA buffer (Beyotime, #P0013B), made of 50 mM Tris-HCl (pH 7.4), 1% NP-40, 150 mM NaCl, 1 mM EDTA, and a 1:400 dilution of a mixture of protease inhibitors. Following centrifugation, the supernatants were combined with 50 µL of anti-Flag M2 magnetic beads (Sigma-Aldrich, #M8823) or anti-HA magnetic beads (Thermo Fisher, #88837) at 4°C for 12 hours. Subsequently, the beads were washed three times in washing buffer [PBS supplemented with 10 µM phenylmethylsulfonyl fluoride (PMSF)] and collected. The precipitated proteins were analyzed by western blot using specified antibodies.

### Western blot analysis

Proteins extracted from cellular lysates were separated by electrophoresis on 8%, 10%, or 12% Bis-Tris SDS-PAGE gels at a fixed pH of 6.4. Subsequently, the resolved protein bands were transferred onto polyvinylidene fluoride membranes. These membranes were then blocked using a 3% bovine serum albumin (BSA) solution in either PBS with Tween-20 (PBST) or Tris-buffered saline with Tween-20 (TBST)—depending on whether phosphorylated proteins were detected—and incubated for 2 hours. Following the blocking step, the membranes were incubated overnight with specific primary antibodies at 4°C. Afterward, the membranes were thoroughly washed with PBST or TBST and then incubated with horseradish peroxidase (HRP)-conjugated secondary antibodies diluted in a 3% BSA solution in PBST or TBST for 1 hour at room temperature. Finally, the protein signals were visualized using Chemistar High-sig ECL western blot substrate (Tanon, #34580) and the Tanon 5200 detection system.

### Enzyme-linked immunosorbent assay

IL-1β in the supernatants of *Zbp1*^+/+^ and *Zbp1*^−/−^ BMDM cultures upon infection with various WT or recombinant viruses was measured by enzyme-linked immunosorbent assay (ELISA) (#ab241673, Abcam) according to the manufacturer’s instruction.

### Hematoxylin and eosin staining

Tissues were fixed in 4% paraformaldehyde for a period exceeding 24 hours, after which they were dehydrated in solutions with an increasing concentration of ethanol. The tissues were then incubated in a 1:1 vol ratio solution of xylene and anhydrous ethanol for 5 minutes, followed by a 10-minute immersion in pure xylene and another 10-minute immersion in renewed pure xylene solution. Subsequently, the specimens were embedded in paraffin, then sectioned, and stained with hematoxylin and eosin (H&E) according to the standard protocol. Finally, the slides were examined using a light microscope.

### Viral infection

Cells were infected with viruses at the specified MOI. Following 2-hour adsorption, the monolayers were overlaid with DMEM supplemented with 2% FBS and then incubated at 37°C. To determine viral titers, samples were collected at 48 hpi, and the viruses, released by three freezing/thawing cycles, were titrated on Vero cells using the plaque assay method. For western blot assay, cell lysates were harvested at the designated timepoints and analyzed.

### Statistical analysis

All data are presented as means ± SD and analyzed using GraphPad Prism 8.0 software. The statistical significance of the results was assessed by one-way Analysis of Variance (ANOVA) with Dunnett’s multiple comparisons or by unpaired two-tailed Student’s t test, as indicated in the figure legends. *P* values were calculated from three biological replicates unless otherwise indicated in the legends. Kaplan–Meier survival curve was assessed by Log-rank test. Data were reproduced in independent experiments as indicated in the figure legends.

## SUPPLEMENTAL MATERIAL

Supplemental figures mbio.01945-24-s0001.docx Figures S1 to S6.

Table S1 mbio.01945-24-s0002.docx Primers.

ASM does not own the copyrights to Supplemental Material that may be linked to, or accessed through, an article. The authors have granted ASM a non-exclusive, world-wide license to publish the Supplemental Material files. Please contact the corresponding author directly for reuse.
