# Supplementary material for: Suppression of ZBP1-mediated NLRP3 inflammasome by the tegument protein VP22 facilitates pseudorabies virus infection
Source: mBio. 2024 Oct 30;15(12):e01945-24. doi: 10.1128/mbio.01945-24 (PMC11633114; doi:10.1128/mbio.01945-24)
Supplement: Supplemental figures — Figures S1 to S6. [file mbio.01945-24-s0001.docx]

**Supplemental Figures**


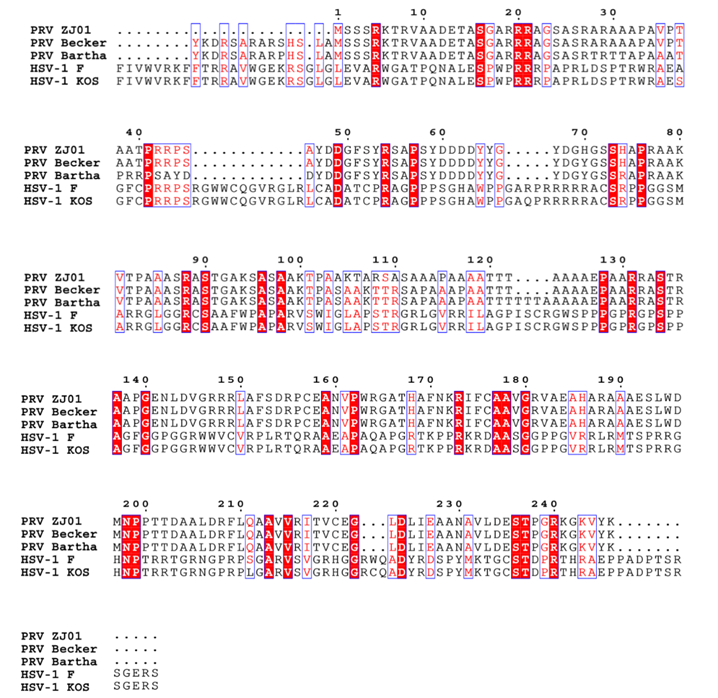


**Fig S1. Sequence homologies between VP22 proteins from PRV and HSV-1.** Alignment of VP22 protein sequences from PRV ZJ01 (GenBank: KM061380.1), PRV Becker (GenBank: JF797219.1), and PRV Bartha (GenBank: JF797217.1) strains, and HSV-1 F (GenBank: GU734771.1) and HSV-1 KOS (GenBank: JQ673480.1) strains, performed with ESPript 3.0 (<https://espript.ibcp.fr/ESPript/ESPript/index.php>). Red boxes show conserved aa between both viruses, while blue boxes show conservative substitutions.


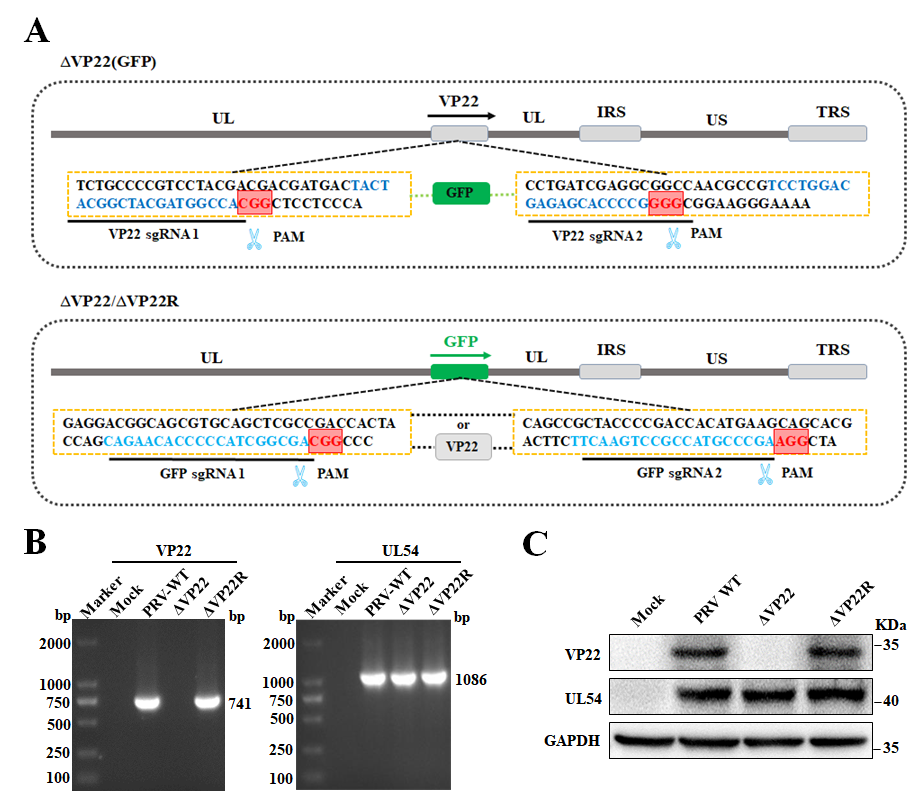


**Fig S2. Graphic representation of recombinant PRV viruses depleted for VP22 or repaired.** (A) Strategy used for PRV-ΔVP22 construction with the CRISPR-Cas9 system; the VP22 locus was initially substituted with a gene encoding the Green Fluorescent Protein (GFP), which was next excised (PRV-ΔVP22), or replaced back with a VP22 gene (PRV-ΔVP22R). (B) Vero cells were mock-infected or infected with either PRV-WT, PRV-ΔVP22 or PRV-ΔVP22R at 5 MOI; at 12 hpi, the cells and supernatants were harvested for viral DNA extraction and PCR detection of the gene fragments encoding respectively, PRV VP22 and UL54, this early gene serving as positive control of virus presence. (C) Vero cells were mock-infected or infected with either PRV-WT, PRV-ΔVP22 or PRV-ΔVP22R at 5 MOI; at 12 hpi, cells were harvested for western blot analysis with antibodies against VP22, UL54 and GAPDH.


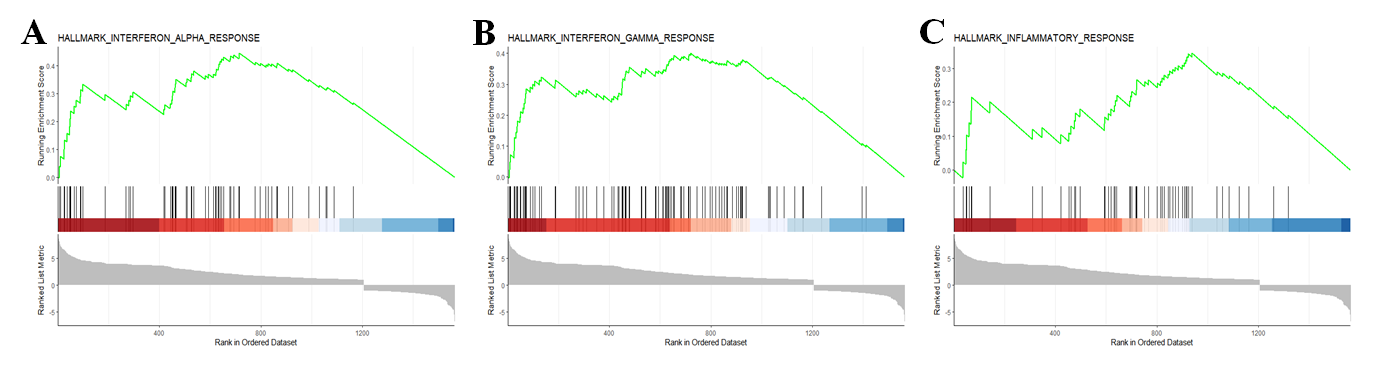


**Fig S3. Gene set enrichment analysis (GSEA) of differentially expressed genes between ΔVP22 versus PRV-WT-infected MEFs.** Findings pertaining to the three pathways related to innate immune responses: (A) IFN-α signaling (B) IFN-γ signaling, and (C) inflammation, among the top six enriched pathways.


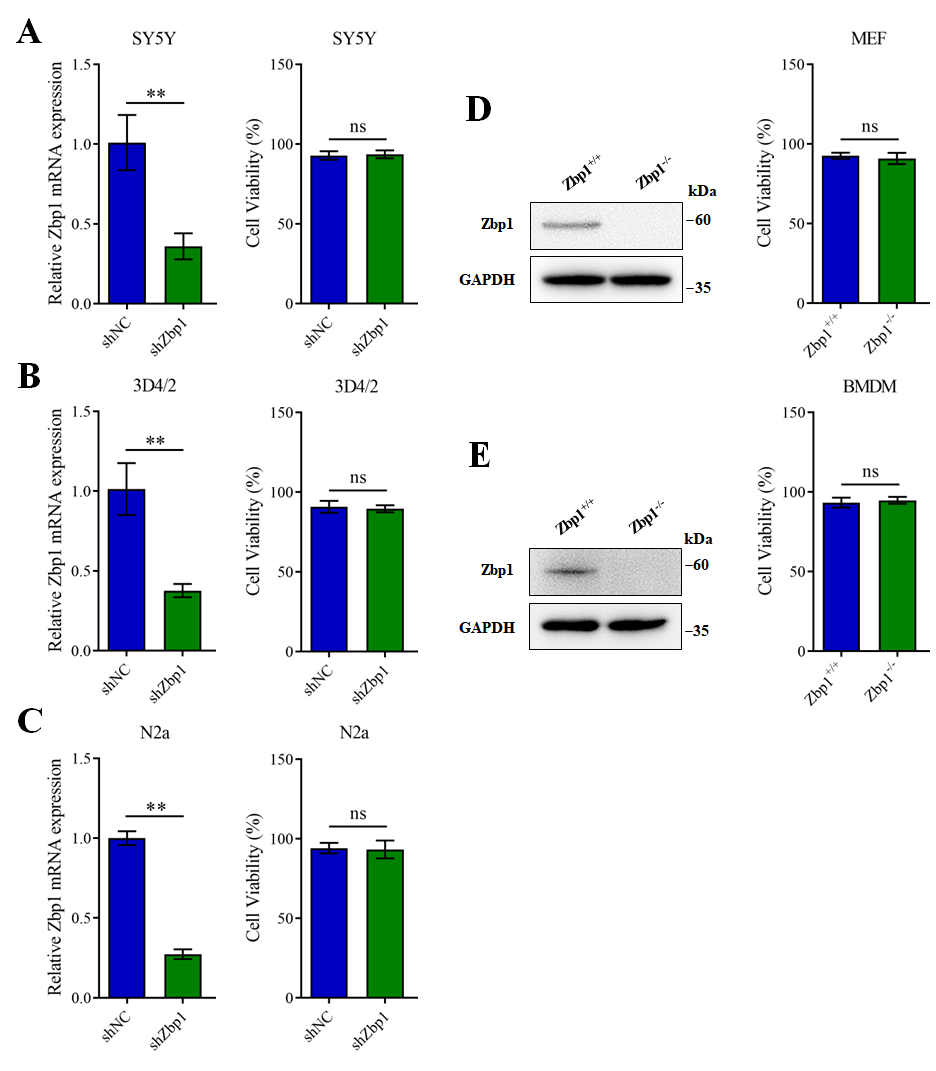


**Fig S4. Evaluation of ZBP1-knockdown efficiency.** (A-C) Detection of relative *Zbp1* mRNA level and cell viability in SY5Y (A), 3D4/2 (B), and N2a (C) cells transduced with control shNC or shZbp1 interfering RNAs. (D and E) Detection of Zbp1 protein expression levels in MEF (D) and BMDM (E) cells isolated from wild-type mice and ZBP1-deficient mice.


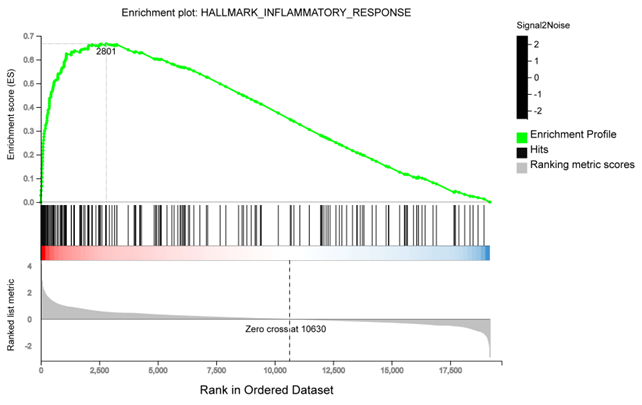


**Fig S5. Gene set enrichment analysis (GSEA) of differentially expressed genes between WT and *Zbp1*-deficient BMDMs infected with PRV-ΔVP22.** Findings pertaining to inflammatory response signaling pathways.


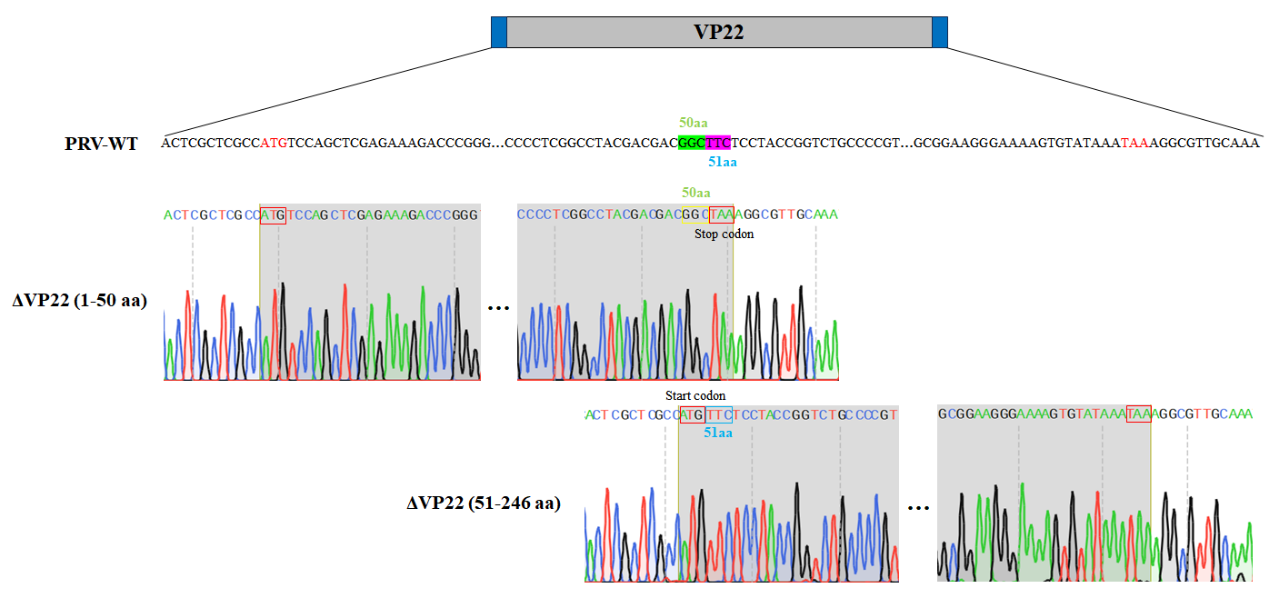


**Fig S6. Validation of engineered** **PRV VP22 (1-50aa) and PRV VP22 (51-246aa) mutant viruses by sequencing.**
