# Supplementary material for: Suppression of ZBP1-mediated NLRP3 inflammasome by the tegument protein VP22 facilitates pseudorabies virus infection
Source: mBio. 2024 Oct 30;15(12):e01945-24. doi: 10.1128/mbio.01945-24 (PMC11633114; doi:10.1128/mbio.01945-24)
Supplement: Table S1 — Primers. [file mbio.01945-24-s0002.docx]

**Table S1. Primers used in this study**

| **Primer** | **Sequence (5′→3′)** |
| --- | --- |
| **Primers used for RT-PCR** | |
| mDdx58-qF | GCAGGTTACTGTGGACTTTGTG |
| mDdx58-qR | TGCCATTCTCCCTTTAGTGTCT |
| mDhx58-qF | TCATCTGTACGGCAGAGTTGT |
| mDhx58-qR | TGTTGTAGACGGTGTCCTTGT |
| mIfih1-qF | GCCTGGAACGTAGACGACAT |
| mIfih1-qR | TGGTTGGGCCACTTCCATTT |
| mTlr3-qF | TCACTTGCTCATTCTCCCTT |
| mTlr3-qR | GACCTCTCCATTCCTGGC |
| mZbp1-qF | GACGACAGCCAAAGAAGTGA |
| mZbp1-qR | GAGCTATGTCTTGGCCTTCC |
| mNlrp3-qF | ATTACCCGCCCGAGAAAGG |
| mNlrp3-qR | TCGCAGCAAAGATCCACACAG |
| mIl-1β-qF | TGTAATGAAAGACGGCACACC |
| mIl-1β-qR | TCTTCTTTGGGTATTGCTTGG |
| RNA18S/Rna18s-qF | CCTGCGGCTTAATTTGACTC |
| RNA18S/Rna18s-qR | AACCAGACAAATCGCTCCAC |
| PRV gB-qF | GTCCGTGAAGCGGTTCGTGAT |
| PRV gB-qR | ACAAGTTCAAGGCCCACATCTAC |
| **Primers used for recombinant PRV** | |
| VP22 sgRNA1-F | CACCGTACTACGGCTACGATGGCCA |
| VP22 sgRNA1-R | AAACTGGCCATCGTAGCCGTAGTAC |
| VP22 sgRNA2-F | CACCGTCCTGGACGAGAGCACCCCG |
| VP22 sgRNA2-R | AAACCGGGGTGCTCTCGTCCAGGAC |
| GFP sgRNA1-F | CACCGCAGAACACCCCCATCGGCGA |
| GFP sgRNA1-R | AAACTCGCCGATGGGGGTGTTCTGC |
| GFP sgRNA2-F | CACCGTTCAAGTCCGCCATGCCCGA |
| GFP sgRNA2-R | AAACTCGGGCATGGCGGACTTGAAC |
| VP22-Left-F | TCTAAGCTTGGAAGACGACGATGCCGCGGAGGTT |
| VP22-Left-R | TATCTGCAGGGCGAGCGAGTGGGATCGGGCGCGT |
| VP22-Right-F | TATGGTACCAGGCGTTGCAAATGGCACCCCGCTC |
| VP22-Right-R | TATGAATTCGCCGGCACCGGCGCGCCCGCCAGCA |
| VP22-CMV-GFP-F | CCGATCCCACTCGCTCGCCCTGCAGGTGATGCGGTTTTGGCAGTACATCAATGGG |
| VP22-SV40-GFP-R | GGTGCCATTTGCAACGCCTGGTACCAACTTGTTTATTGCAGCTTATAATGGTT |
| VP22-ΔVP22R-F | CCGATCCCACTCGCTCGCCCTGCAGATGTCCAGCTCGAGAAAGACCCGGGTCGCCGCC |
| VP22-ΔVP22R-R | GGTGCCATTTGCAACGCCTGGTACCTTATTTATACACTTTTCCCTTCCGCCCCG |
| VP22-ΔVP22 (1-50 aa)-F | CCGATCCCACTCGCTCGCCCTGCAGATGTCCAGCTCGAGAAAGACCCGGGTCGCCGCC |
| VP22-ΔVP22 (1-50 aa)-R | GGTGCCATTTGCAACGCCTGGTACCTTAGCCGTCGTCGTAGGCCGAGGGGCGTCT |
| VP22-ΔVP22 (51-246 aa)-F | CCGATCCCACTCGCTCGCCCTGCAGATGTTCTCCTACCGGTCTGCCCCGTCCTACGAC |
| VP22-ΔVP22 (51-246 aa)-R | GGTGCCATTTGCAACGCCTGGTACCTTATTTATACACTTTTCCCTTCCGCCCCG |
| VP22-ΔVP22-F | TCTCTTCCTCCGCCCGGCTCTCGCTGAC |
| VP22-ΔVP22-R | GGGCAGCTCCTCCTCCACGGCCGGGACC |
| PRV VP22-F | ATGTCCAGCTCGAGAAAGACCCGGGT |
| PRV VP22-R | TTATTTATACACTTTTCCCTTCCGCC |
| PRV UL54-F | ATGGAGGACAGCGGCAACAGCAGCGGCA |
| PRV UL54-R | TCAAAACAGGTGGTTGCAGTAAAAGTAC |
| **shRNA sequences** | |
| shmZBP1-F | CCGGGCGATTATTTGTCAGCACAATCTCGAGATTGTGCTGACAAATAATCGCTTTTTG |
| shmZBP1-R | AATTCAAAAAGCGATTATTTGTCAGCACAATCTCGAGATTGTGCTGACAAATAATCGC |
| shhZBP1-F | CCGGGCACAATCCAATCAACATGATCTCGAGATCATGTTGATTGGATTGTGCTTTTTG |
| shhZBP1-R | AATTCAAAAAGCACAATCCAATCAACATGATCTCGAGATCATGTTGATTGGATTGTGC |
| shsZBP1-F | CCGGCTGCCCAGCTGGTCATCAAATCTCGAGATTTGATGACCAGCTGGGCAGTTTTTG |
| shsZBP1-R | AATTCAAAAACTGCCCAGCTGGTCATCAAATCTCGAGATTTGATGACCAGCTGGGCAG |
| **Primers used for plasmid construction** | |
| pCMV-HA-ZBP1(whole)-F | GCAGGATCCATGGCAGAAGCTCCTGTTGACTTGA |
| pCMV-HA-ZBP1(whole)-R | GCACTCGAGTCATTGCTTGCTCAGTCCTGTGTCT |
| pCMV-HA-ZBP1(C domain)-F | ATAGGATCCATGGATGCAGGGAAGCACCCCTCTTATA |
| pCMV-HA-ZBP1(C domain)-R | GCACTCGAGTCATTGCTTGCTCAGTCCTGTGTCT |
| pCMV-HA-ZBP1(ΔZα)-F | GCAGGATCCATGCAGGAAGGCCAAGACATAGCTCATT |
| pCMV-HA-ZBP1(ΔZα)-R | GCACTCGAGTCATTGCTTGCTCAGTCCTGTGTCT |
| pCMV-HA-ZBP1(Zα)-F | GCAGGATCCATGGCAGAAGCTCCTGTTGACTTGA |
| pCMV-HA-ZBP1(Zα)-R | GCACTCGAGTCAATTGGCAATGGAGATGTGGCTGTTG |
| pCMV-N-Flag-VP22(whole)-F | AATGGATCCATGTCCAGCTCGAGAAAGACCCGGG |
| pCMV-N-Flag-VP22(whole)-R | GCACTCGAGTTATTTATACACTTTTCCCTTCCGC |
| pCMV-N-Flag-VP22(50-246 aa)-F | ATAGGATCCATGGGCTTCTCCTACCGGTCTGCCCCGT |
| pCMV-N-Flag-VP22(50-246 aa)-R | GCACTCGAGTTATTTATACACTTTTCCCTTCCGC |
| pCMV-N-Flag-VP22(100-246 aa)-F | ATAGGATCCATGAAGACCCCCGCGGCCAAGACCGCCC |
| pCMV-N-Flag-VP22(100-246 aa)-R | GCACTCGAGTTATTTATACACTTTTCCCTTCCGC |
| pCMV-N-Flag-VP22(50-246 aa)-F | AATGGATCCATGTCCAGCTCGAGAAAGACCCGGG |
| pCMV-N-Flag-VP22(50-246 aa)-R | ATACTCGAGTCACGGGGGGTTCATGTCCCAGAGGGAC |
| pCMV-N-Flag-GFP-F | CCGAATTCATGGTGAGCAAGGGCGAGGAGCTGTT |
| pCMV-N-Flag-GFP-R | AACTCGAGTTACTTGTACAGCTCGTCCATGCCGA |
| pCMV-HA-GFP-F | CCGAATTCATGGTGAGCAAGGGCGAGGAGCTGTT |
| pCMV-HA-GFP-R | AACTCGAGTTACTTGTACAGCTCGTCCATGCCGA |
| pCMV-N-MYC-RIPK3-F | GCCAAGCTTATGTCTTCTGTCAAGTTATGGCCTA |
| pCMV-N-MYC-RIPK3-R | AATCTCGAGCTACTTGTGGAAGGGCTGCCAGCCCCTACCCC |
| F, forward; R, reverse | |
